# Compliance with Smoke-Free Policy and Challenges in Implementation: Evidence from Bengkulu, Indonesia

**DOI:** 10.31557/APJCP.2020.21.9.2647

**Published:** 2020-09

**Authors:** Sepri Yunarman, Agustin Zarkani, Ahmad Walid, Abdillah Ahsan, Dian Kusuma

**Affiliations:** 1 *Faculty of Tarbiyah and Tadris, Institut Agama Islam Negeri Bengkulu, Bengkulu, Indonesia. *; 2 *Faculty of Agriculture, University of Bengkulu, Bengkulu, Indonesia. *; 3 *Faculty of Economics and Business, Universitas Indonesia, Depok, Indonesia. *; 4 *Centre for Health Economics and Policy Innovation, Imperial College Business School, London, UK. *

**Keywords:** Tobacco control, smoke-free policy, compliance, challenge, Indonesia, Bengkulu

## Abstract

**Background:**

Smoking is among the top contributors to deaths and disability-adjusted life years in Indonesia, particularly among males. In 2012, a presidential decree encouraged provinces and districts to implement a smoke-free policy (SFP). This study aims to evaluate compliance and explore the challenges in the implementation.

**Methods:**

Through a mixed-methods design, we used quantitative methods to examine the compliance with six criteria including signage, no active smoking, no selling, no advertisement, no smoke, and no ashtray at SFP facilities in Bengkulu city. We observed SFP compliance at 105 facilities, including health/educational facilities, places of worship, workplaces, and indoor/outdoor public facilities. We also used a qualitative method to explore challenges in the implementation through interviews with the government and legislators.

**Results:**

The compliance rate to all six criteria was 38% overall, ranging from 17% at outdoor public facilities to 67% at health facilities. We found no spatial patterning, as shown by non-significant differences in compliance rates between SFP facilities inside and outside of 1-kilometer around the provincial and city health offices. Implementation challenges included lack of sensitization, lack of coordination, and limited budget.

**Conclusion:**

The compliance was relatively low due to several challenges, which could serve as a tobacco control policy lesson in a lower-middle-income country.

## Introduction

Cardiovascular diseases contributed to 353 million disability-adjusted life years globally in 2016, of which 44% were attributed to smoking (Gakidou et al., 2017; Naghavi et al., 2017). Similarly, smoking was among the top contributors to disability-adjusted life years in Indonesia in 2017, particularly among males (Mboi et al., 2018). Smoking prevalence among men (15+ years) is among the highest in the world at 67%, while that among boys (13-14 years) is high at 36.2% (Ministry of Health, 2011; World Health Organization, 2018).

The smoke-free policy (SFP), recommended by the Framework Convention on Tobacco Control, has been among the most effective policy against smoking. Previous studies showed that it is associated with reduced smoking rates in the United States, reduced indoor smoking in the United Kingdom, lowered secondhand smoke exposure in New Zealand, reduced myocardial mortality in Belgium, and increased indoor air quality in 15 countries in North America and Europe (Connolly et al., 2009; Cox et al., 2014; Edwards et al., 2008; Hahn et al., 2008; Lee et al., 2011). In Indonesia, two regulations related to SFP are Health Act 36/2009 (President of Indonesia, 2009) and Presidential Decree 109/2012 (President of Indonesia, 2012). While the Act provided a recommendation to implement SFP in all provinces and cities/districts, the Decree provided the details. The law prohibits producing, selling, advertising, promotion, and smoking of tobacco products at SFP facilities, which include health facilities and schools. However, only two-thirds of districts (345 of 514 areas) have enacted some form of SFP by December 2018 (Wahidin et al., 2020), with significant variations in compliance from 17% in Jayapura city in 2018 to 78% in Bogor city in 2011 (Asyary and Veruswati, 2018; Wahyuti et al., 2019). 

Bengkulu city, the capital of Bengkulu province in the Sumatera region, has an estimated population of 368 thousand in 2017 (Statistics Bureau, 2018). The city adopted SFP through the Local Bill 3/2015, which the province did through the Local Bill 4/2017. The Bills banned selling, advertising, promotion, and smoking of tobacco products in various facilities, including health facility, educational facility, place of worship, office, and indoor public facilities, and outdoor public facilities. There are, however, criticisms on the lack of enforcement of the policy, including at government offices and schools (Azhar, 2018). Our study is to provide evidence on the compliance with and challenges in SFP implementation in Bengkulu city, which is currently lacking. 

## Materials and Methods

Through a mixed-methods design, we used both quantitative and qualitative methods. The quantitative approach was to assess the compliance to six SFP criteria, including “no smoking” signage, no smoking activity, no selling, no adverts, no cigarette smoke, and no ashtray. The facility groups were health facilities such as hospitals, clinics, and pharmacies; educational facilities such as kindergarten, primary schools, high schools, and universities; places of worship such as mosques, churches, and temples; workplaces such as government offices and private company offices; indoor public facilities such as public transport vehicles, malls, hotels, restaurants, and child play station; and outdoor public facilities such as public parks. Because of limited resources, we purposive sampled 105 facilities, including each facility group (see Table 1). At each facility, we observed the SFP criteria and interviewed respondents (e.g., facility owners, managers, sellers, and religious leaders) on their knowledge and perceptions. The qualitative approach explored the challenges through in-depth interviews with four key stakeholders from the governments (governor’s office, provincial health office, and civil police office) and local parliament. Six trained research assistants conducted the data collection from October to December 2019. 

**Figure 1:**
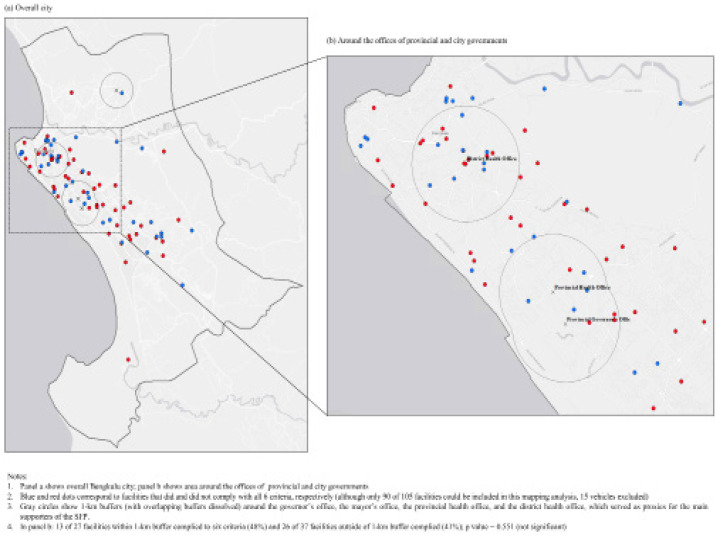
Maping Facilities that Did and Did not Comply with the Smoke-Free Policy in the City of Bengkulu, 2019

For data analyses, we conducted a descriptive result in STATA 15.1 to examine the compliance rates at all facilities and each group. We also did a spatial analysis in ArcMap 10.6 to explore any spatial patterning in compliance with SFP. In ArcMap, we used the buffer tool to generate facility buffer (1-kilometer, about 15-minute walk) around four facilities: the governor’s office, mayor’s office, provincial health office, and district health office. Because they should be the main supporters of SFP, we hypothesized for higher compliance among facilities inside the buffer (Figure 1). We collected data on geolocations of the facilities using Google MyMaps (post survey). Finally, we conducted a thematic content analysis to elicit implementation challenges.

Ethical clearance was from the University of Hasanuddin Faculty of Public Health (No. 999/UN4.14.7.1/TP.01.02/2020).

## Results

Table 1 shows a total sample of 105 facilities including 15 (14%) health facility, 19 (18%) educational facility, 15 (14%) places of worship, 15 (14%) workplaces, 35 (33%) indoor public facilities, and 6 (6%) outdoor public facilities. Among all facilities, the overall compliance with six criteria was 38%, ranging from 57% compliance with having “no smoking” signage to 96% compliance with no adverts. Notably, the overall compliance with no advertisement was very high at 89% among indoor public facilities and 100% among health facilities, education facilities, places of worship, workplaces, and outdoor public facility. Similarly, the compliance rates with no selling were high, ranging from 83% among outdoor public facilities to 100% among health facilities and educational facilities. The compliance rates with no active smoking were high among most facility groups (80% to 93%), except among outdoor public facilities (33%). 

Among health facilities, 73% of clinics/pharmacies complied with six criteria, while only 50% of hospitals did. Among educational facilities, 67% of universities met all six criteria, while only 63% of kindergarten/primary schools and high schools did. Among places of worship, 33% of churches complied with all criteria, while only 11% of mosques did (mainly due to violation with no signage, for both facilities). Among workplaces, only 33% of government offices, government-owned company offices, and private company officers complied with all criteria. Among indoor public facilities, 67% of malls and cinemas met all six criteria, while only none of the hotels did. Among outdoor public facilities, only 17% of public parks complied with all criteria. 

Table 2 shows the knowledge and perception of SFP at the facilities among respondents (facility owners, managers, sellers, and religious leaders). Only 50% of respondents reported knowing about the SFP law (mostly knowledge that SFP is a public health effort against smoking and that one can smoke only in designated areas). Moreover, 71% of respondents reported their facility had a smoke-free rule, and 84% of them said they informed employees and or visitors about the smoke-free effort. However, only 31% reported had no difficulties in keeping their facility smoke free, including being ignored and objection. By facility group, 93% of respondents at health facilities and workplaces said their facility had a smoke-free rule, but only 47% of them at places of worship did.

Figure 1 shows the mapping of the facilities to explore any spatial patterning of compliance. Panel a shows the overall city map, and panel b shows the area around government offices (governor’s and health offices). Blue and red dots show facilities that did and did not comply, respectively (only 90 of 105 facilities were used because 15 vehicles could not be mapped). Grey circles show a 1-kilometer (dissolved) buffer around the government offices as a proxy for the main supporters of SFP. Results show slightly higher compliance rates with six criteria among facilities inside the buffer (48%, 13 complied out of 27 facilities) compared to those outside (41%, 26 complied out of 37 facilities); but the difference was not statistically significant (p-value = 0.551). 

Moreover, results from the content analysis show three main challenges in the SFP implementation: lack of sensitization, lack of coordination, and limited budget. Activities to raise awareness by the Provincial Health Office as the initiator were limited to stickers at the entrance of government office and banners on the streets on harmful effects and “no smoking” warning. There have not been any sensitization activities directly to government offices or community groups. Also, there were only limited coordination activities among key stakeholders. Informants from the provincial offices mentioned the difficulty of implementing the SFP was because the cities/districts are decentralized and report directly to the Ministry of Home Affairs in Jakarta. Third, informants have also raised concerns on the limited budget. The civil police, who is responsible for enforcing the implementation through field visit, will start to have funding for monitoring and enforcement in 2020. The local parliament currently has no funding for SFP monitoring because of other priorities such as infrastructure and agriculture. 

**Table 1:** Compliance (n, %) with the Smoke Free Policy by Facility Type in Bengkulu City, 2019

	Total	Signage		No smoking		No sale		No advert		No smoke		No ashtrays		Compliance all 6	
(a) Overall	105	60	57%	89	85%	95	90%	101	96%	91	87%	94	90%	40	38%
(b) Group															
Health facility	15	11	73%	14	93%	15	100%	15	100%	14	93%	15	100%	10	67%
Educational facility	19	13	68%	16	84%	19	100%	19	100%	18	95%	18	95%	12	63%
Place of worship	15	4	27%	14	93%	13	87%	15	100%	14	93%	15	100%	3	20%
Workplace	15	10	67%	12	80%	13	87%	15	100%	12	80%	12	80%	5	33%
Public facility indoor	35	19	54%	31	89%	30	86%	31	89%	31	89%	28	80%	9	26%
Public facility outdoor	6	3	50%	2	33%	5	83%	6	100%	2	33%	6	100%	1	17%
(c) Facility															
Health															
Hospitals	4	3	75%	3	75%	4	100%	4	100%	3	75%	4	100%	2	50%
Clinics/pharmacy	11	8	73%	11	100%	11	100%	11	100%	11	100%	11	100%	8	73%
Education															
Kinder/primary	8	6	75%	7	88%	8	100%	8	100%	8	100%	8	100%	5	63%
High school	8	5	63%	6	75%	8	100%	8	100%	7	88%	7	88%	5	63%
University	3	2	67%	3	100%	3	100%	3	100%	3	100%	3	100%	2	67%
Place of worship															
Mosque	9	1	11%	9	100%	7	78%	9	100%	9	100%	9	100%	1	11%
Church/temple	6	3	50%	5	83%	6	100%	6	100%	5	83%	6	100%	2	33%
Workplace															
Government office	9	7	78%	6	67%	7	78%	9	100%	6	67%	7	78%	3	33%
Govt-owned company	3	1	33%	3	100%	3	100%	3	100%	3	100%	3	100%	1	33%
Private company	3	2	67%	3	100%	3	100%	3	100%	3	100%	2	67%	1	33%
Public indoor															
Public transport vehicle	14	2	14%	14	100%	14	100%	14	100%	13	93%	14	100%	1	7%
Mall/cinema	3	3	100%	3	100%	3	100%	2	67%	3	100%	3	100%	2	67%
Hotel	3	3	100%	1	33%	1	33%	2	67%	2	67%	0	0%	0	0%
Cafe, restaurant, store	6	4	67%	6	100%	3	50%	4	67%	6	100%	3	50%	1	17%
Child play station, park	9	7	78%	7	78%	9	100%	9	100%	7	78%	8	89%	5	56%
Public outdoor															
Public parks	6	3	50%	2	33%	5	83%	6	100%	2	33%	6	100%	1	17%

Note: Total, sample (purposive); %, proportion; No smoking, no smoking activity; no smoke, no cigarette smoke; Clinics include government and private clinics. Public transport vehicle includes bus and taxis. Indoor public parks include museum. Outdoor public parks include one motorcycle taxi. All 6 comply, comply with all six criteria (signage, smoking, selling, advert, smoke, and ashtray).

**Table 2:** Knowledge and Perception (n, %) on SFP at Facility

	Total	Knew about the SFP law		Facility had smoke-free rule		Ever informed employee/visitor about smoke-free facility		Had no difficulty in keeping the facility smoke free	
(a) Overall	105	53	50%	75	71%	88	84%	26	31%
(b) Group									
Health facility	15	9	60%	14	93%	15	100%	3	20%
Education facility	19	11	58%	14	74%	17	89%	5	29%
Place of worship	15	11	73%	7	47%	15	100%	4	29%
Workplace	15	12	80%	14	93%	14	93%	5	42%
Public facility indoor	35	9	26%	22	63%	22	63%	5	25%
Public facility outdoor	6	1	17%	4	67%	5	83%	4	80%

Note: Respondents included facility owners, managers, sellers, and priests. Knowledge about the law also included knowledge that SFP is a public health effort against smoking and that one can smoke only in designated areas. Activities to inform employee or visitors included telling not to smoke, having no-smoking signage, and providing smoking area. Difficulties in keeping the facility smoke free included being ignored and objection.

## Discussion

We found an overall SFP compliance of 38% in Bengkulu city. This evidence should raise concern among policymakers given that the policy was enacted since 2015 by the city bill and again since 2017 by the provincial law. While this rate is higher than that in Jayapura city (17%), it is much lower than that in Bogor city (78%) and Punjab, India (84%) (Asyary and Veruswati, 2018; Goel et al., 2018; Wahyuti et al., 2019). By SFP criteria, however, we found that the compliance rates with no advertising, no selling, or no active smoking were relatively high in most facility groups, ranging from 80% to 100%. The only exception is the compliance to no active smoking among outdoor public parks that was very low at 33%. This is encouraging for potential reduction of smoking, indoor smoking, and exposure to secondhand smoke at SFP facilities in the city (Connolly et al., 2009; Edwards et al., 2008; Hahn et al., 2008). 

Moreover, the compliance rates were highest among health facilities (67%) and educational facilities (63%). These rates are higher compared to the overall compliance (38%) and the compliance among health and educational facilities in Jayapura city (50% and 29%, respectively) (Wahyuti et al., 2019). These rates, however, are much lower compared to the compliance rates among health and educational facilities in Punjab, India (90% and 85%, respectively) (Goel et al., 2018). The compliance rates among places of worship and workplaces were lower (33%) than the overall compliance rate (38%). This should also of concern for policymakers because places of worship such as mosques are regularly in use by community members, including children for daily prayers and after-school Quran classes. The lower compliance at workplaces should also alert the stakeholders because increased exposure to secondhand smoke through poorer air quality (Connolly et al., 2009). Previous studies also showed that smoke-free workplaces are associated with smoke-free homes in India and Nigeria (Kaleta, 2015; Lee et al., 2014), another much-needed initiative in Indonesia (Trisnowati et al. 2019). The lowest compliance rate was among outdoor public parks in the city (17%). Concerted efforts to increase smoke-free public parks even by only using signage are needed to help reduce smoking use (Platter and Pokorny, 2018).

All this evidence should trigger action from the local and national authorities if tobacco control efforts are to be successful. Political support and allocated budget are needed to have better sensitization, coordination, and enforcement efforts. Our study, however, has limitations in terms of sampling and areas. Further SFP assessment should employ random and more representative sampling of not only urban areas (e.g., provincial capital) but also rural areas (e.g., rural districts).

## Funding

Support was provided by the Center for Islamic Economics and Business, Universitas Indonesia, with funding awarded by Bloomberg Philanthropies to Johns Hopkins University. Its content is solely the responsibility of the authors and do not necessarily represent the official views of Bloomberg Philanthropies or Johns Hopkins University.
